# What happened to parents’ views of school success for autistic children during the COVID-19 pandemic?

**DOI:** 10.3389/fpsyt.2023.1211041

**Published:** 2023-08-24

**Authors:** Sheng-Li Cheng, Sanyin Cheng, Shushan Liu, Yun Li

**Affiliations:** School of Philosophy and Social Development, Shandong University, Jinan, Shandong, China

**Keywords:** views of school success, pandemic stress, parental involvement, autistic children, implications for future

## Abstract

**Background:**

The educational views of parents with autistic children directly impacts their children’s academic success. However, little research has been done on how the COVID-19 pandemic impacted parents’ academic and social views.

**Aim:**

This study analyzes parents’ views of school success for their autistic children in the context of the COVID-19 pandemic and examines the relationships among pandemic stress, parental involvement, and parents’ views of school success for autistic children in mainland China.

**Methods:**

In this study, 713 parents of autistic children completed measures assessing their pandemic stress, parental involvement, and views of school success; linear regression and structural equation modeling were used to analyze the data.

**Results:**

Parents’ views of school success were influenced by factors such as parents’ level of education, household income, parents’ gender, and children’s age. The effects of pandemic stress on views of school success for parents of autistic children are complex: *physical and mental reaction* has a negative direct effect on *views of school success*, a positive indirect effect mediated by *parental involvement*, and a net positive effect; *risk perception and concern* has a negative indirect effect; and both the direct and indirect effects of *pragmatic hopefulness* are positive. Education policymakers and practitioners need to seriously and carefully assess these results’ implications for modern, inclusive education.

## 1. Introduction

Not only has the pandemic seriously affected people’s daily lives and behaviors, including those of parents of autistic children, but the pandemic stress has also affected parents’ views of school success for their children. Pandemic stress means individuals’ feelings of stress related to COVID-19. Parents’ views of school success herein indicate whether parents believe the child is doing well.

Autism is a neurodevelopmental disorder that affects an individual’s abilities in social interaction, communication, interests, and behavior (1). Parents of autistic children are often subjected to many stressors and challenges that may affect their emotional and psychological well-being and expectations for their children’s future education (2, 3). The uncertainty and risks associated with the pandemic have increased the burden and stress on parents and may increase their concerns about their children’s health and future (4, 5). Although previous studies have demonstrated changes in views of school success among parents of deaf or hard-of-hearing children during a pandemic (6, 7), fewer studies have examined, from a more systematic perspective, the impact of pandemic stress on views of school success of parents of autistic children. Our study provides new findings to explore the relationships among pandemic stress, parental involvement, and parents’ views of school success in autistic children in mainland China.

### 1.1. Studies on parents’ views of school success for autistic children

Scholars in China have reached a consensus regarding parents’ views of school success, including their beliefs on talent, parenting, child development, and education (8, 9). In China, parents’ views of school success have evolved from a traditional view of school success emphasizing intelligence and high expectations to a new view of school success focusing on modern social skills and good quality (10, 11). Factors such as parental educational attainment (11, 12), family income (13), and occupation (12) all contribute to shaping parents’ views of school success. Notably, since the outbreak of COVID-19, parents’ educational expectations, influenced by factors such as distance learning and segregation, have also transitioned from a focus on schoolwork and extracurricular activities to a focus on the development of their children’s livelihood skills and an expectation that their children will become more independent (14).

Research on parents’ views of school success of children with disabilities originated from Westling (15) study, which suggested that parents are both partners in the educational process and consumers of schools and institutions. Parents of autistic children experience greater stress than parents of children with other disabilities (16), highlighting the importance of considering their views of school success.

Existing research on parents of autistic children’s views of school success consists of three main categories. The first one concerns studying the impact of parents’ views of school success on their autistic children, with almost all studies confirming they can be important in predicting achievement for youth with disabilities, including autism (17–19). The second describes parents’ goals related to their views of school success for autistic children, including increased expectations that their autistic children will find work, live independently (20), have contact with normally-developing children (21), and have the highest possible level of education and success in school (22, 23). The third explores what influences parents of autistic children’s views of school success, including parental socioeconomic status (18, 19), educational attainment (24), and the extent of their children’s problematic behaviors (24, 25). Bush et al. (24) found that male parents had higher expectations for autistic children than female parents. Thomas et al. (25) found that among 24 parents of autistic children, higher parental psychopathology and autism severity were associated with lower expectations. More recent studies have also demonstrated that stress induced by COVID-19 negatively predicts views of school success among parents of children with disabilities (6, 18).

Most studies have centered on characterizing parents of autistic children’s views of school success. While some studies have investigated the factors influencing these perspectives, few have delved into the specific mechanisms through which stress affects parents’ views of school success. Despite the passage of time since the onset of the COVID-19 pandemic, its repercussions persist, underscoring the significance of examining the influence of COVID-19-related stress on parents’ views of school success for autistic children. Therefore, further research is warranted to elucidate the interplay between stress and parents’ views of school success for autistic children in light of the ongoing impact of the pandemic.

### 1.2. Psychological stress related to autism and its association with their views of school success

During the COVID-19 pandemic, the government implemented a policy of isolating individuals in their homes to prevent the spread of the virus (26). While this policy effectively reduced the spread of the pandemic, it also had unintended consequences, such as increased anxiety, depression, and psychological stress among individuals (27–29). Parents who were raising infants and children with disabilities affected by the pandemic also received less social support and experienced higher levels of psychological stress (30–33).

Studies have shown that parents of autistic children experienced even higher levels of psychological stress during the pandemic (34) and that this stress profoundly impacted their expectations of and planning for their children’s education (35, 36). As we move into the post-pandemic era, exploring the relationship between psychological stress and views of school success is of great academic interest. Understanding this relationship can help parents of autistic children reduce psychological stress and improve their views of school success. As a result, this area of research has received increasing attention (2, 6).

The study of parenting stress among parents of autistic children and their views of school success can be divided into three main aspects. The first describes the current state of parents’ psychological stress and their views on school success. Due to their child’s autism, these parents experience higher levels of psychological stress, and their views on school success are generally lower than those of parents of children without autism (19, 37).

In the second aspect, parents’ views on school success are used as a factor to assess their psychological stress outcomes. Several studies have used the extent of their views on school success as a measure of parents of autistic children’s psychological stress (38–40).

The third aspect involves analyzing the causal relationship between parents’ psychological stress and their views of school success. For example, Cheng and Cheng (6) found that psychological stress caused by COVID-19 significantly negatively predicted views of school success among 213 parents of deaf children. Similarly, Cheng et al. (2) found that psychological stress caused by COVID-19 significantly negatively predicted views of school success among 1919 parents of children with developmental disabilities. Thomas et al. (25) found that higher parental psychopathology and autism severity were both related to lower expectations among 24 parents of children diagnosed with autism.

Existing research has made progress in exploring the relationship between parental psychological stress and school success perceptions, confirming a negative correlation between them (7, 41). However, it is important to note that the impact of parental psychological stress on views of school success is not a simple linear relationship, but a complex dynamic process, particularly during the pandemic. To the best of the author’s knowledge, no studies have investigated the mechanisms by which pandemic stress affects parents’ views of school success among parents of autistic children as a group. Therefore, this study explores the enduring mechanisms of COVID-19 pandemic stress to identify and discuss its role in shaping parents of autistic children’s views of school success.

### 1.3. Parental involvement as an intermediary

The focus on parental involvement originated in a study by Englund et al. (42), which noted a positive correlation between parental involvement and children’s school performance. Existing research has sorted the main elements and common forms of parental involvement into two main areas. The first is educational expectations, parenting styles, and educational career planning for their children (43–45). The second is interaction with organizations such as schools, communication followed by parenting plans, and children working together to complete parental involvement plans (46, 47).

The treatment and developmental process of autistic children requires full parental involvement; therefore, it is important to study parental involvement and its influencing factors. Caring for autistic children requires more time and energy, and parent education and medical interventions are particularly critical in autistic children’s early development years (48, 49). Parents are involved in all aspects and critical periods of their child with autism’s development, including participating in the child’s development, learning, and treatment and actively communicating with teachers and physicians (50, 51). At the same time, parents of autistic children become involved in their child’s academic life and rehabilitation process, such as tutoring their child’s homework, participating with them in interest classes, and designing and intervening in their child’s rehabilitation program (28, 52–54). Involvement in their child’s development, learning, and treatment can help parents fully understand their child’s daily life, using their views of school success as an indicator, and help them develop a clearer perception of the efficiency of behaviors and the feasibility of programs relating better to their child (55, 56).

Partial researches suggest that parental involvement positively influences behaviors, habits, academic performance, social performance, and future planning in autistic children (57–59). Existing research has focused on the negative predictive effects of parental stress on parental involvement in autistic children, showing that higher levels of parental stress will result in reduced parental involvement and supportive behaviors (60, 61).

Results of related studies have shown a significant correlation between parental stress and parental involvement (62, 63), while other studies have demonstrated that parental involvement can influence their views of school success (64, 65). Several studies conducted with Chinese autistic children and their parents have shown that the parents have higher levels of psychological stress than parents of typically-developing children or children with other types of disabilities, negatively affecting their level of parental involvement (66, 67). Therefore, exploring whether parental involvement mediates the relationship between pandemic stress and views of school success is critical.

### 1.4. The present research

This study aimed to investigate the relationships among pandemic stress, parental involvement, and their views of school success for parents of autistic children. Three research questions were proposed:

(1) What is the current status of parents’ views of school success for autistic children in China in the context of the COVID-19 pandemic?

(2) How do demographic variables, such as gender, education, household income, etc., influence parents’ views of school success for autistic children?

(3) What are the relationships among pandemic stress, parental involvement, and parents’ views of school success?

Based on previous studies [e.g., (2, 24, 25)], it was hypothesized that:

(1) Parents’ demographic variables (e.g., gender, education, and income) would predict their views of school success for their children; and

(2) Pandemic stress would directly predict their views of school success but indirectly predict their views of school success, mediated by parental involvement.

**Figure 1:**
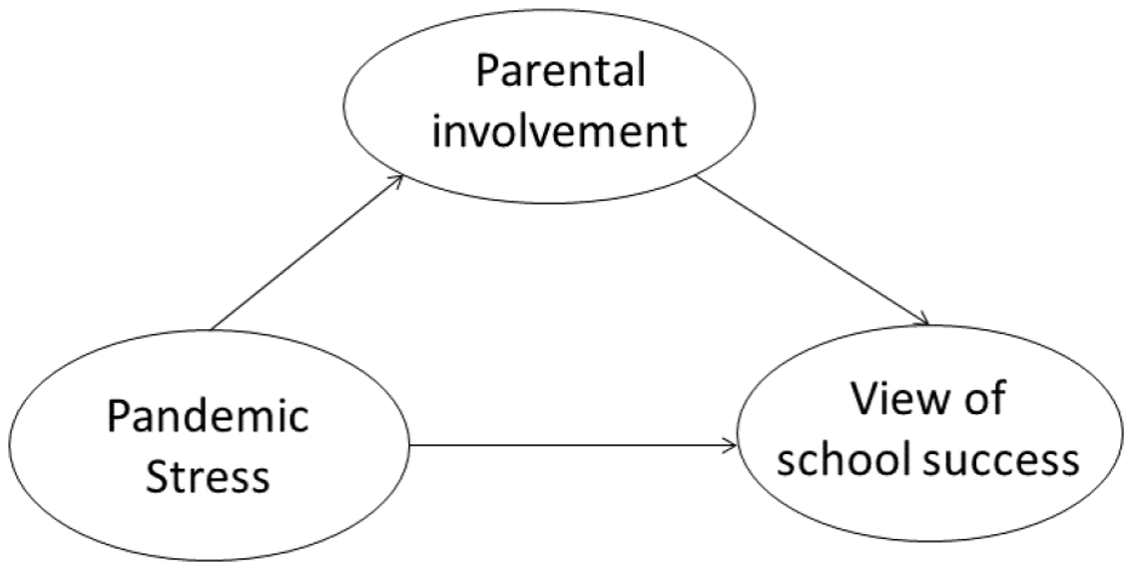
Hypothetical model.

The hypothetical model proposed in this study is shown in Figure 1.

## 2. Methods

### 2.1. Procedures

This study was conducted using an online survey due to time and geographical limitations and because direct contact with parents of autistic children was not feasible. In October 2020, the survey was presented to the headmasters of public schools offering special services to autistic children younger than 22 in 31 provinces, autonomous regions, and municipalities, who then forwarded the survey to parents. In the accompanying letter, we assured parents that their responses would be kept confidential and used solely for academic research purposes and that participation was voluntary. The online survey was designed to take approximately 20 min; after completion, a token of appreciation was sent to participating parents in the form of a random bonus pack.

### 2.2. Participants

A total of 801 parents of autistic children completed the questionnaire. Considering that autistic children tend to lag behind normal children in development and growth, the study used being younger than 22 years as a screening condition to remove outliers; the final sample size was 713. As shown in Table 1, 16% of participants were males and 84% were females, mostly between the ages of 31 and 50. Nearly 40% of the participants held a bachelor’s degree or above. Most families had a monthly income of less than 10,000 yuan (equivalent to US$1500). Most of the autistic children were younger than 17, and close to 40% were primary school students.

**Table 1:** Participant descriptive statistics.

Variables	n	%
Parent		
Gender		
Male	113	15.8%
Female	600	84.2%
Age		
18–25	6	0.8%
26–30	33	4.6%
31–40	332	46.6%
41–50	292	41.0%
51–60	41	5.8%
>60	9	1.3%
Education
<High school	99	13.9%
Polytechnic school or high school	122	17.1%
Mechanical degree or bachelor degree	198	27.8%
>Bachelor degree	294	41.2%
Monthly household income (Yuan)		
<5K	269	37.7%
5K–10K	233	32.7%
10K–20K	115	16.1%
>20K	96	13.5%
Number of children		
1	365	51.2%
2	319	44.7%
3	29	4.1%
Children		
Age		
<7	231	32.4%
8–17	408	57.2%
18–22	74	10.4%
Education		
Not enrolled	141	19.8%
Kindergarten	108	15.1%
Primary schools	310	43.5%
Junior high school	92	12.9%
Polytechnic school or high school	54	7.6%
Specialist or undergraduate	8	1.1%

### 2.3. Measures

A demographic sheet and three scales were adopted in this study. The demographic sheet included questions to gather personal information about the respondents (i.e., gender, age, employment status, educational level, family structure, monthly household income, number of children) and their children with disabilities (i.e., age, educational level). The three scales were as follows.

#### 2.3.1. Academic and social success scale

The academic and social success scale (68) is an 18-item self-report scale that examines parents’ views of their children’s school success on a five-point Likert-type scale (1 = very unsuitable, 2 = unsuitable, 3 = neither suitable nor suitable, 4 = suitable, 5 = very suitable).

The scale was developed in English, translated into Chinese for the research conducted in China, and then back-translated into English. It has two factors: (1) academic success, which assesses how important it is for parents to have their child succeed academically (10 items); a sample item is “How important is it for you that your child learn to speak English?”; and (2) social success, which assesses how important the child’s social success is to the parent (eight items); a sample item is “How important is it to you that your child gets along well with classmates?”

Considering the potential for localization in categorizing the scale’s items, after removing 48 outliers, the researchers conducted an exploratory factor analysis (EFA) of the scale for optimization.

Factor analysis is appropriate when the KMO (Kaiser-Meyer-Olkin) > 0.60 and Bartlett’s spherical test is statistically significant (69). Results showed that KMO = 0.93 and Bartlett sig < 0.05, indicating that the scale was suitable for EFA.

The rotated component matrix shows that there are still two factors in the scale which can still be named academic success and social success; however, the items in the two factors are different from the original scale. Three items previously belonging to academic success now become items of social success, which are “How important it is to you that your child follows the teacher’s instructions,” “How important it is to you that your child follows school rules,” and “How important it is to you that your child enjoy school.” In Chinese culture, they are more like the social aspect of school success. One item previously belonging to social success became an item of academic success: “How important is it to you that your children learn to get along with people from different cultures.” For Chinese parents, it is more like an academic aspect of school success because they may relate “getting along with people from different cultures” to “learning to speak a foreign language.” Thus, there were eight items in academic success and 10 in social success, different from the original scale’s 10 academic success and eight social success items.

**Figure 2:**
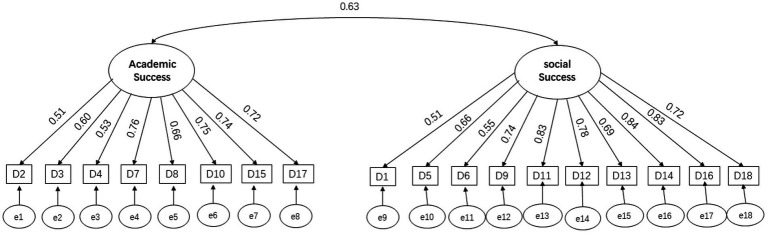
Confirmatory factor analysis of the academic and social success.

The study validated the modified scale by confirmatory factor analysis (CFA), and the reliability and validity of the scale met the requirements of psychometric indicators and showed good reliability and validity. As shown in Figure 2, the overall Cronbach’s alpha value of the scale in this study was 0.91, the alpha value of academic success was 0.87, and the alpha value of social success was 0.91, indicating a good level of internal consistency of the whole scale and the two factors. The CFA results of the scale showed that CMIN/DF was 4.68, RMSEA (Root Mean Square Error of Approximation) was 0.07, CFI (Comparative Fit index) was 0.94, AGFI (Adjusted Goodness-of-fit index) was 0.88, IFI (Incremental Fit index) was 0.94, and TLI (Tucker-Lewis index) was 0.92, all of which were good indicators. Compared with the scale designed by Ryan, the modified scale has better fit and more desirable data.

#### 2.3.2. Pandemic stress scale

This study uses the Pandemic Stress Scale (5) to measure stress resulting from experiencing the COVID-19 pandemic. This measurement tool employs a five-point Likert-type scale and was developed by Wang et al. (26), who designed nine items divided into three factors. Cheng et al. further improved the original scale by redefining the factors through exploratory factor analysis (EFA) and a component matrix. The first factor, *Pragmatic Hopefulness* (two items), reflects the perceived status of parents of autistic children and their confidence in the COVID-19 pandemic. The second factor, *Risk Perception and Concern* (five items), reflects respondents’ perceived risk and concern about their and their family members’ infection. The third factor, *Physical and Mental Reaction* (two items), reflects a range of physical and mental reactions and changes during the COVID-19 pandemic. The Cronbach’s alpha value for the scale was 0.77, indicating good internal consistency.

#### 2.3.3. Parental involvement scale

This study localized the Parental Involvement Scale (2022) by Cheng et al. to the Chinese context and tailored it to the study’s purpose. The scale consists of 24 items, divided into four factors, each containing six items. The first factor, *Involvement in School Activities*, reflects how closely participants interact with their child’s school. The second factor, *Anxiety and Overprotection,* reveal specific behaviors of anxiety and overprotection in child-rearing. The third factor, *Homework Help*, reflects subjects’ involvement in their child’s classroom tutoring and academic development. The fourth factor, *Interest Development-Extracurricular Activities,* reflects participants’ involvement in their child’s hobbies and interests. The Cronbach’s alpha values were 0.90 for the scale and 0.81, 0.76, 0.85, and 0.85 for the four subscales, indicating good internal consistency across the survey instrument and the four subscales. The indicators of the scale met the criteria for good model plot fit, including a CMIN/DF of 3.19, RMSEA of 0.06, GFI of 0.92, CFI of 0.93, AGFI of 0.90, IFI of 0.93, and TLI of 0.92. These measures indicate that the scale has good reliability and validity and meets the psychometric indicators’ requirements.

### 2.4. Data analysis

We analyzed the data using SPSS 27.0, Stata 16.0, and Amos 28.0. As the survey was conducted online and only complete answers could be submitted, there were no missing values. We processed outliers using SPSS27.0, removing 48 samples with standard scores greater than or less than 3, leaving a final sample of 713. An examination of correlations revealed that no independent variables were highly correlated (*r* > 0.80). The multi-collinearity statistics, including Tolerance and VIF (Variance Inflation Factor), were within acceptable limits.

An exploratory factor analysis of the Academic and Social Success Scale was conducted using SPSS 27.0, and the scale was adjusted and validated using AMOS 28.0. Following this, SPSS 27.0 was used to describe each subscale of the three scales to explore the current state of parental views of school success, pandemic stress, and parental involvement at this stage. A one-sample t-test was conducted to assess the mean difference between participants’ perceptions of these variables and the hypothesized midpoint score (i.e., critical value = 3). In addition, regression analyses were conducted on two subscales of the Academic and Social Success Scale using Stata 16.0 to explore the effects of demographic variables on different school success orientations.

**Figure 3:**
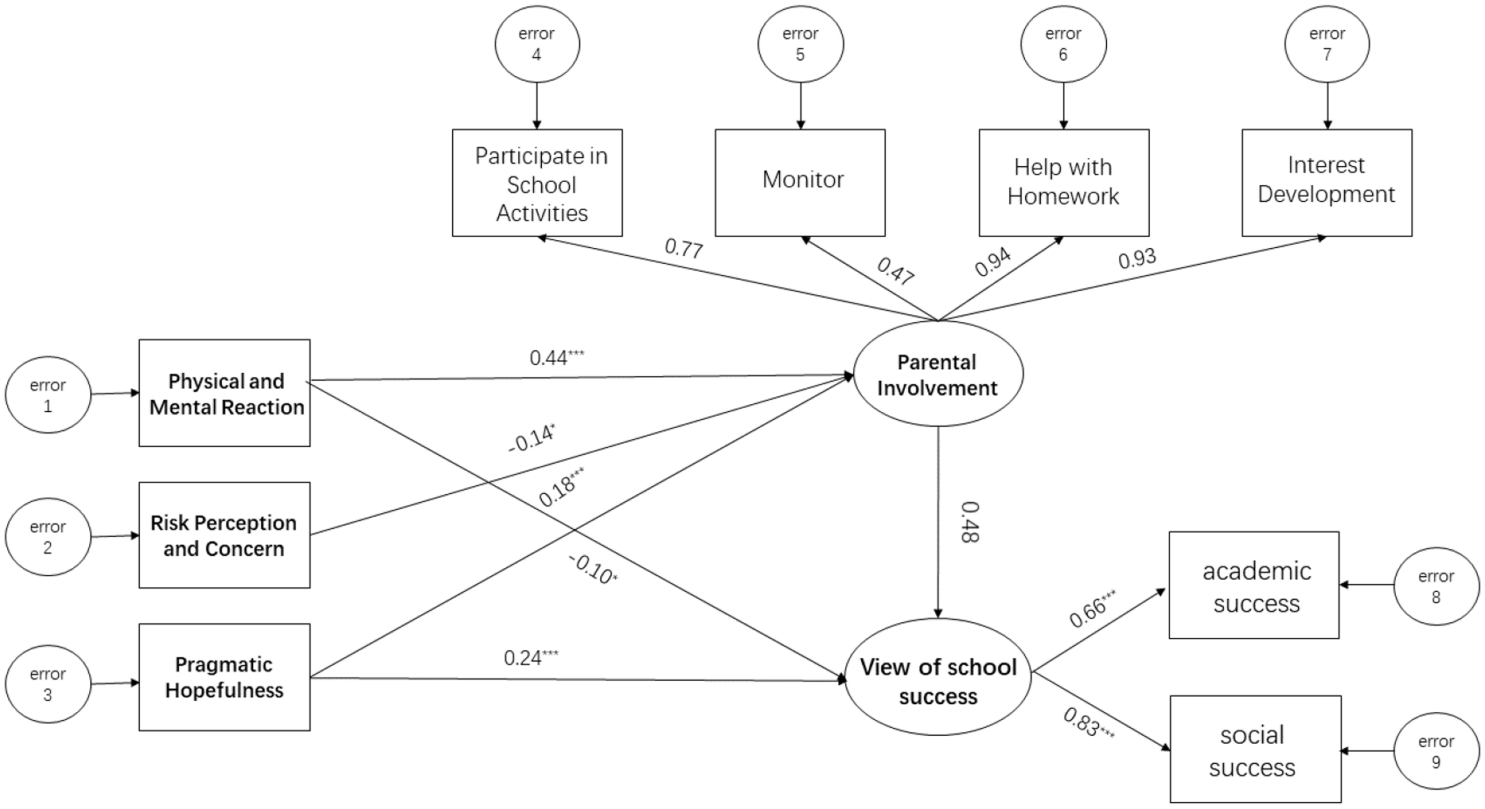
Structural equation model path diagram of the interrelations between stress, involvement, and views of school success. ^*^p < 0.05, ^***^p < 0.001.

Structural equation modeling (SEM) has been applied to test hypothetical models (see Figure 1). Structural equation modeling is a series of multivariate statistical models used to estimate the effects and relationships between multiple variables representing a theoretical model of a hypothesis (70). In this study, the three variables were the sum of the items from each scale. Due to the low loading coefficients of the *risk perception and concern* and *physical and mental reaction* subscales on pandemic stress, a new model was reconstructed in which the three factors of pandemic stress were used as the observed independent variables (see Figure 3).

## 3. Results

### 3.1. Descriptive results

The first research question addressed the current status of pandemic stress, parental involvement, and their views of school success among families of autistic children in China. As shown in Table 2, academic success [*M* = 3.36, *SD* = 0.76, *t*(712) = 12.54, *p* < 0.001] and social success [*M* = 4.23, *SD* = 0.59, *t*(712) = 56.15, *p* < 0.001] were significantly higher than the expected average (score 3), and social success was significantly higher than academic success [*t*(712) = 72.69, *p* < 0.001].

**Table 2:** Descriptive statistics for family quality of life, parental involvement, and stress.

	n	m	sd	df	T	*p*
AS	713	3.36	0.76	712	12.54	<0.001
SS	713	4.23	0.59	712	56.15	<0.001
PS	713	3.78	0.68	712	30.90	<0.001
PM	713	3.70	0.64	712	29.11	<0.001
PH	713	3.90	0.67	712	35.71	<0.001
PI	713	4.04	0.62	712	44.82	<0.001
EW	713	2.50	0.91	712	−14.68	<0.001
EE	713	2.84	0.83	712	−5.18	<0.001
EP	713	3.92	0.65	712	37.77	<0.001

AS, academic success; SS, social success; PS, participate in school activities; PM, monitor; PH, help with homework; PI, interest development; EE, physical and mental reaction; EW, risk perception and concern; EP, pragmatic hopefulness.

Parents’ involvement with their autistic children was above average (score 3). *Interest development* was highest [*M* = 4.04, *SD* = 0.62, *t*(712) = 44.82, *p* < 0.001], followed by *help with homework* [*M* = 3.90, *SD* = 0.67, *t*(712) = 35.71, *p* < 0.001], *monitor* was lowest [*M* = 3,70, *SD* = 0.64, *t*(712) = 29.10, *p* < 0.001], followed by *participate in school activities* [*M* = 3.78, *SD* = 0.68, *t*(712) = 30.90, *p* < 0.001].

Pandemic stress was above our expected average (score 3). *Risk perception and concern* [*M* = 2.50, *SD* = 0.91, *t*(712) = −14.68, *p* < 0.001] and *physical and mental reaction* [*M* = 2.84, *SD* = 0.83, *t*(712) = −5.18, *p* < 0.001] were lower than the expected mean, while *pragmatic hopefulness* was higher [*M* = 3.92, *SD* = 0.65, *t*(712) = 37.77, *p* < 0.001].

### 3.2. Multiple regression analyses results

Multiple regression models were used to test the first hypothesis—that parents’ gender, education, income, etc., will predict their views of school success for their children. The model results are detailed in Table 3. As shown in Model 1, the following options—gender (*p* < 0.1), 26–30 years old (*p* < 0.05), 31–40 years old (*p* < 0.01), 41–50 years old (*p* < 0.01), 51–60 years old (*p* < 0.05), older than 60 (*p* < 0.05), monthly household income of $10,000–20,000 (*p* < 0.1), >$20,000 (*p* < 0.05), being an only child (*p* < 0.05), age of the child with autism being 8–17 years old (*p* < 0.01) and 18–22 years (*p* < 0.1), the grade level of the child with autism being primary school (*p* < 0.01), junior high school (*p* < 0.01), and polytechnic school or high school (*p* < 0.01)—were significantly associated with academic success. The effects between the other options for the other variables and academic success were not significant.

**Table 3:** Predicting parents’ view of children’s school success from their demographic background information.

	Model 1	Model 2
	Academic success	Social success
Parent		
Male	0.172^+^	−0.202^*^
	(0.100)	(0.091)
Age		
26–30	−1.049^*^	−0.586
	(0.442)	(0.403)
31–40	−1.192^**^	−0.856^*^
	(0.408)	(0.372)
41–50	−1.109^**^	−0.705^+^
	(0.402)	(0.366)
51–60	−0.970^*^	−0.706^+^
	(0.418)	(0.381)
>60	−1.060^*^	−0.601
	(0.506)	(0.462)
Education		
Polytechnic school or high school	0.167	0.182
	(0.131)	(0.119)
Mechanical degree or bachelor degree	0.074	0.102
	(0.123)	(0.112)
>Bachelor degree	0.055	0.302^**^
	(0.127)	(0.116)
Monthly household income (Yuan)		
5K–10K	−0.128	−0.146^+^
	(0.089)	(0.081)
10K–20K	−0.223^+^	−0.085
	(0.117)	(0.107)
>20 K	−0.277^*^	−0.000
	(0.125)	(0.114)
Children		
Only child	−0.179^*^	−0.167^*^
	(0.072)	(0.066)
Age		
8–17	−0.283^**^	−0.216^*^
	(0.107)	(0.097)
18–22	−0.355^+^	−0.008
	(0.183)	(0.167)
Education		
Kindergarten	0.114	0.320^**^
	(0.128)	(0.116)
Primary schools	0.289^**^	0.437^***^
	(0.108)	(0.099)
Junior high school	0.383^**^	0.338^*^
	(0.144)	(0.131)
Polytechnic school or high school	0.509^**^	0.422^*^
	(0.189)	(0.172)
Specialist or undergraduate	0.566	−0.182
	(0.382)	(0.348)
_cons	1.246^**^	0.629^+^
	(0.418)	(0.381)
N	713	713
*R* ^2^	0.055	0.081

Standard errors are in parentheses. ^+^*p* < 0.10, ^*^p < 0.05, ^**^p < 0.01, ^***^p < 0.001.

As shown in Model 2, the following options—gender (*p* < 0.05), 31–40 years old (*p* < 0.05), 41–50 years old (*p* < 0.1), 51–60 years old (*p* < 0.1), bachelor’s degree education or higher (*p* < 0.01), monthly household income of $5,000–10,000 (*p* < 0.1), being an only child (*p* < 0.05), age of the child with autism being 8–17 years old (*p* < 0.05), the grade level of the child with autism was kindergarten (*p* < 0.01), primary school (*p* < 0.001), junior high school (*p* < 0.05), and polytechnic school or high school (*p* < 0.05)—were significantly associated with social success. The effects between other options and social success for the other variables were not significant. Based on the results of the multiple regression analysis, the first hypothesis was fully tested.

### 3.3. SEM results

The third research question concerned the relationship between views of school success, parental involvement, and pandemic stress. The model fit indices indicate that the proposed model was adequate, Where X^2^/df = 14.85, RMSEA = 0.14, GFI = 0.90, AGFI = 0.82, CFI = 0.70, IFI = 0.73, TLI = 0.61. The modified index function was used to see if Amos 28.0 suggested additional improvements to the model. After modification, the SEM model fit index was better, where X^2^/df = 5.13, RMSEA = 0.07, GFI = 0.97, AGFI = 0.93, CFI = 0.94, IFI = 0.94, and TLI = 0.88, all of which are excellent levels. In addition, through model exploration with Amos 28.0, the output suggested removing the direct covariate pathway between views of school success and *risk perception and concern*. We decided to retain the modified model (see Figure 3), with the following interpretation of the results.

The standardized loadings for each latent variable ranged from 0.66 to 0.83 for views of school success and 0.47–0.94 for parental involvement, with standardized loadings for all latent variables exceeding the critical value of 0.40, indicating that the observed subscales adequately measured the latent variables.

SEM results showed a direct negative effect of *physical and mental reaction* on views of school success, with an effect size of −0.10, and an indirect positive effect on views of school success through the mediating variable *parental involvement,* with a size of 0.44*0.48 = 0.211. The total effect of *physical and mental reaction* on views of school success was positive, with a size of 0.211–0.1 = 0.111. The mediating variable of parental involvement fully mediated the relationship between *risk perception and concern* and views of school success. This effect size was −0.14*0.48 = −0.067, indicating that *risk perception and concern* had a negative impact on views of school success that was partially explained by parental involvement. *Pragmatic hopefulness* had a direct positive effect on views of school success of size 0.24 and an indirect positive effect on *views of school success* through the mediating variable parental involvement of size 0.18*0.48 = 0.086. The total effect of *pragmatic hopefulness* on views of school success is positive, with a size of 0.24 + 0.086 = 0.326 (see Table 4).

**Table 4:** Results of structural equation model analysis.

Model	EE	EW	EP	Involvement
Direct effects				
View of school success	−0.10*		0.24***	0.48***
Indirect effects				
Involvement	0.44***	−0.14***	0.18***	
View of school success	0.211***	−0.067***	0.086***	
Total				
View of school success	0.111*	−0.067***	0.326***	

EE, physical and mental reaction; EW, risk perception and concern; EP, pragmatic hopefulness. *p < 0.05, ***p < 0.001.

Overall, the model results support the second hypothesis. Pandemic stress would predict their views of school success, directly and indirectly, mediated by parental involvement. Specifically, the pandemic latent variable *pragmatic hopefulness* had a direct positive effect on views of school success, while *physical and mental reaction* had a direct negative effect. All three latent pandemic stress variables indirectly affected views of school success. *Physical and mental reaction* and *pragmatic hopefulness* were positively associated with views of school success through parental involvement, while *risk perception and concern* was negatively associated.

## 4. Discussion

This study aimed to describe the current status of views of school success, parental involvement, and pandemic stress among parents of autistic children, the demographic factors influencing parental views of school success among parents of autistic children, and to explore the relationship between parental views of the school, parental involvement, and pandemic stress. The results responded well to the research questions, and the research hypotheses were largely supported.

### 4.1. The current status of parental views of school success and the impact of demographic variables

The findings showed that parents scored higher on their children’s social achievement orientation, answering the first research question, and that parents of autistic children’s views of school success were significantly influenced by their education level, whether they were only children, their family income, and their children’s grade levels, answering the first research question and confirming the first research hypothesis.

First, the descriptive results showed that parents of autistic children scored higher on social achievement orientation than on academic success orientation. On the one hand, this may be because Chinese parents’ views of school success are moving in a scientific and rational direction, with academic success not being the only criterion by which parents judge their children (11, 71); on the other hand, parents of autistic children have lower expectations of their children’s school success, preferring that their children communicate well with others and engage in some degree of community involvement (21, 72). Therefore, parents of autistic children have higher levels of their children’s social success than their children’s academic success.

Second, we performed a multiple regression using Stata 16.0 to answer the second research question. Based on the results of multiple regressions, parents’ views of school success were significantly influenced by demographic variables, including parents’ education level, whether they were only children, family income, and their children’s grade level, consistent with the results of previous studies. The research found that parents’ having bachelor’s degrees and above was positively associated with their views of social success for autistic children, indicating parents with higher education levels had more rational and scientific educational attitudes, in line with the trend of Chinese parents’ positive scientific attitudes toward education (11, 71). In addition, this study found that fathers were more inclined to expect their children’s academic success than mothers, highly consistent with previous findings (24). Meanwhile, parents with only one child had significantly lower views of school success than parents with multiple children. This may be due to the increased stress on parents during COVID-19 when only children are more in need of parental protection and care, which in turn lowers expectations for their children. Finally, the social success of children not enrolled in school was lower. We speculate that this may be due to these children’s younger age and their parents’ greater focus on basic life skills than social success.

### 4.2. The relationships among pandemic stress, parental involvement, and their views of school success

The study’s results showed that parental involvement behaviors positively predicted their views of school success, validating the research hypothesis and consistent with current research (24, 55). Parents of autistic children are actively involved in their child’s school, life, and rehabilitation. This behavior will positively influence parents’ views of their children’s school success, including academic success and social success.

SEM results showed that the *physical and mental reaction* had a direct negative predictive effect on parents’ views of school success. The indirect positive predictive effect of *physical and mental reaction* on parents’ views of school success was mediated by parental involvement. Overall, *physical and mental reaction* had a positive predictive effect on parents’ views of school success. This is not entirely consistent with previous findings (6) because this study included parental involvement as a mediator in the model. During the COVID-19 pandemic, factors such as government quarantine policies and the risk of contracting the pandemic disease placed people at higher levels of *physical and mental reaction* (32); Parents’ views of school success assessed the importance of their children’s academic and social success to parents, and *physical and mental reaction* during the pandemic placed parents at higher stress levels, creating a sense of urgency that contributed to higher levels of stress. *Physical and mental reaction* during the pandemic placed parents at higher stress levels, and the resulting sense of urgency promoted increased parental involvement (5), thus positively enhancing their views of school success.

*Pragmatic hopefulness* had a direct positive predictive effect on parents’ views of school success. Meanwhile, mediated by parental involvement, *pragmatic hopefulness* indirectly positively predicted their views of school success. Thus, parents’ *pragmatic hopefulness* for autistic children positively influenced their views on school success. The higher the level of *pragmatic hopefulness*, the higher the level of parental involvement. At the same time, the increased level of parental involvement increased their views of school success. Families of autistic children had financial difficulties and psychological stress due to the COVID-19 pandemic, and maintaining rational judgment and pragmatic hope about the pandemic was an important factor in changing the status quo. Raising the level of *pragmatic hopefulness* among parents of autistic children requires the attention and support of the government and society. On the one hand, schools and medical institutions should help families of autistic children to establish rational and optimistic perceptions about the pandemic, learn to self-regulate during the special period, and use their resources rationally (5, 61). On the other hand, public health departments should conduct a series of courses and lectures related to the pandemic to help parents of autistic children acquire knowledge about the pandemic’s impact on their health and their children’s development (73, 74).

Although *risk perception and concern* did not directly predict parents’ views of school success, it did indirectly negatively predict them, mediated by parental involvement. An increase in *risk perception and concern* was followed by a decrease in parental involvement, which was, in turn, followed by a decrease in parents’ views on their children’s academic and social success. *Risk perception and concern* about the COVID-19 pandemic risk may have distracted parents of autistic children from being involved in their children’s learning and lives (5), resulting in lower levels of parental involvement.

## 5. Significance, limitations, and implication

This study is pioneering in exploring the impact of pandemic stress on parents’ views of school success among autistic children in the context of COVID-19. It contributes to the existing research on pandemic stress and validates the mediating role of parental involvement, enriching research on the correlation between parental involvement and views of school success. Second, this study examines the impact of demographic variables on parents’ views of school success for autistic children in the context of pandemic stress. Its findings could be compared longitudinally with pre-pandemic studies in the future. Third, this study updates the scale of views of school success, which we revised based on data collected from parents of autistic children, completing the localization process and improving the scale’s applicability in China.

There are three limitations to the current study. First, there was an imbalance in the ratio of fathers (16%) to mothers (84%) in the sample, which somewhat influenced the results of the gender variable on views of school success. Second, due to the impact of COVID-19, data were collected using an online questionnaire. Parents of autistic children who do not have access to online devices, have low income, and have limited resources were excluded from our sample; thus, the sample’s representativeness needs further validation. Last, this study was cross-sectional; a longitudinal study design could be conducted in the future to better argue for a causal relationship between pandemic stress on parents’ involvement and their views of school success.

The findings of this study offer valuable insights for enhancing parents’ views of school success in the context of COVID-19, specifically for parents of autistic children. By focusing on the three factors of COVID-19 pandemic stress rather than on pandemic stress as a single variable, we could better address the negative impact of COVID-19 on parents of autistic children and the negative effects of COVID-19 pandemic stress on parents’ views of school success.

Higher *risk perception and concern* mean higher anxiety and worry, which itself is negative for parents of autistic children and has a negative indirect impact (mediated by *parental involvement*) on their views of school success for their autistic children, so providing social support services to reduce their anxiety and worry could help them live better and improve their views of school success for their autistic children when there is a new pandemic outbreak.

*Physical and mental reaction* means that parents of autistic children were negatively affected by the COVID-19 pandemic, which also had a negative direct impact and a positive indirect impact on their views of school success for their autistic children. Psychological counseling should be provided for them if they need to lower their physical and mental reaction and improve their daily life during the pandemic, although this might also indirectly lower their views of school success for their autistic children.

*Pragmatic hopefulness* means accepting the reality of the pandemic outbreak but being confident in the success of the anti-epidemic campaign. *Pragmatic hopefulness* can help parents of autistic children remain stable and active during a pandemic and has a positive direct and indirect impact (mediated by *parental involvement*) on their views of school success for their autistic children. So, effective actions taken by the government and society during the pandemic to support parents of autistic children and maintain their *pragmatic hopefulness* can help them cope with the difficulties and challenges brought by the pandemic and improve their views of school success for their autistic children. For example, government and education departments communicate updates and policies about COVID-19 to parents through official channels, such as websites, social media, and television commercials. This can help parents stay up-to-date on what is happening in schools and education during this period, thus reducing their anxiety and uncertainty (75, 76). This might be the most important implication of our research.

Although COVID-19 has ended, its impact on us continues. The impact of pandemic stress on views of school success remains instructive for how we can better enhance the views of school success for parents of autistic children. First, psychological interventions are needed to reduce the stress felt by parents of autistic children. Positive thinking therapy applies to all parents of autistic children, and parents can learn positive meditation and other techniques to reduce their emotional stress. Second, parents of autistic children need more professional support. Parents can communicate with their child’s education and treatment professionals to better understand their child’s progress in school and treatment and to receive additional guidance and advice. Finally, raising the pragmatic hope of parents of autistic children in the face of crisis events can be achieved through encouragement and guided action. Parents can also be helped to keep up to date with the situation in schools and education, thus reducing their anxiety and uncertainty.

## Data availability statement

The raw data supporting the conclusions of this article will be made available by the authors, without undue reservation.

## Ethics statement

The studies involving human participants were reviewed and approved by the Ethics Committee of Shandong University. The patients/participants provided their written informed consent to participate in this study.

## Author contributions

S-LC supervised the whole manuscript. SC was responsible for the conceptualization of the manuscript. SL and YL were responsible for drafting the manuscript. All authors contributed to the article and approved the submitted version.

## Funding

This manuscript was supported by Shandong Province Taishan Scholar Project Special Fund (No. tsqn202306071) and Analysis of the Path of Social Work Intervention in the Connection of Autistic Children’s Primary Education under the Background of Inclusive Education (No. 11090013552301) supported by Autism Research Special Fund of Zhejiang Foundation For Disabled Persons.

## Conflict of interest

The authors declare that the research was conducted in the absence of any commercial or financial relationships that could be construed as a potential conflict of interest.

## Publisher’s note

All claims expressed in this article are solely those of the authors and do not necessarily represent those of their affiliated organizations, or those of the publisher, the editors and the reviewers. Any product that may be evaluated in this article, or claim that may be made by its manufacturer, is not guaranteed or endorsed by the publisher.
