# Corrigendum: What happened to parents’ views of school success for autistic children during the COVID-19 pandemic?

**DOI:** 10.3389/fpsyt.2024.1384532

**Published:** 2024-03-07

**Authors:** Sheng-Li Cheng, Sanyin Cheng, Shushan Liu, Yun Li

**Affiliations:** School of Philosophy and Social Development, Shandong University, Jinan, Shandong, China

**Keywords:** views of school success, pandemic stress, parental involvement, autistic children, implications for future

In the published article, there was an error in the Funding statement. The correct Funding statement appears below.

This manuscript was supported by Shandong Province Taishan Scholar Project Special Fund (No. tsqn202306071) and Analysis of the Path of Social Work Intervention in the Connection of Autistic Children’s Primary Education under the Background of Inclusive Education (No. 11090013552301) supported by Autism Research Special Fund of Zhejiang Foundation For Disabled Persons.

The authors apologize for this error and state that this does not change the scientific conclusions of the article in any way. The original article has been updated.

